# Co-overexpression of *AVP1* and *OsSIZ1* in Arabidopsis substantially enhances plant tolerance to drought, salt, and heat stresses

**DOI:** 10.1038/s41598-019-44062-0

**Published:** 2019-05-21

**Authors:** Nardana Esmaeili, Xiaojie Yang, Yifan Cai, Li Sun, Xunlu Zhu, Guoxin Shen, Paxton Payton, Weiping Fang, Hong Zhang

**Affiliations:** 1 Department of Biological Sciences, Texas Tech University, Lubbock, Texas 79409 USA; 2 Economic Crop Research Institute, Henan Academy of Agricultural Sciences, Zhengzhou, 450002 China; 3 Zhejiang Academy of Agricultural Sciences, Hangzhou, China; 4 USDA-ARS Cropping Systems Research Laboratory, Lubbock, Texas USA

**Keywords:** Plant stress responses, Molecular engineering in plants

## Abstract

Abiotic stresses such as water deficit, salt, and heat are major environmental factors that negatively affect plant growth, development, and productivity. Previous studies showed that overexpression of the Arabidopsis vacuolar H^+^-pyrophosphatase gene *AVP1* increases salt and water deficit stress tolerance and overexpression of the rice SUMO E3 ligase gene *OsSIZ1* improves heat and water deficit stress tolerance in transgenic plants. In this report, the effects of co-overexpression of *AVP1* and *OsSIZ1* in Arabidopsis on abiotic stress tolerance were studied. It was found that *AVP1/OsSIZ1* co-overexpressing plants performed significantly better than *AVP1*-overexpressing plants and *OsSIZ1*-overexpressing plants, and produced 100% more seed than wild-type plants under single stress or multiple stress conditions. The increased stress tolerance in *AVP1/OsSIZ1* co-overexpressing plants was substantially larger than the increased stress tolerance in *AVP1*-overexpressing plants and *OsSIZ1*-overexpressing plants under every abiotic stress condition tested. This research provides the proof-of-concept that crop yields might be substantially improved using this approach.

## Introduction

Water deficit, salinity, and heat are major environmental factors that negatively affect every aspect of plant growth and development, leading to substantial yield losses in agriculture. World population is growing steadily, and it will likely pass 9 billion in 2050^1^. Direct improvement in crop productivity under limited inputs (primarily water) and marginal growing conditions is critical if we are to meet future demands of global food and fiber production. Irregular rainfall, depletion of groundwater supplies, and competition for freshwater with rapidly-expanding urban areas, all place significant constraints on resources for agricultural productivity. Exacerbating these issues is the fact that the average air temperature is projected to increase 1.5–4 °C by the end of the century and for many regions may result in even more unpredictable weather patterns^2^. It is therefore imperative to develop crops that are more tolerant to drought, heat, and salt stresses, thereby producing sustainable yields under severe stress conditions. Over the last 20 years, many genes have been reported to confer increased stress tolerance when overexpressed in transgenic plants, and some of these genes might be useful in the engineering of crops for higher stress tolerance^3^. However, the successful implication of this strategy in agriculture is currently very limited. The reason was that most studies used single genes in transgenic plants and the increased stress tolerance was not high enough, or the specificity of increased tolerance was limited to one stress in most cases, which did not lead to substantially increased yields under field conditions^3^. In production agriculture, abiotic stresses usually do not occur in isolation; instead, they often occur in combinations. For arid and semi-arid production environments, water deficit stress often occurs during heat waves, and a direct response to water deficit is stomatal closure, which results in elevated leave temperatures and thermal stress^4^. Thus, it is likely necessary to combine the benefits of multiple transgenes to engineer abiotic stress tolerance or improve crop yields under multiple stress conditions.

Salinity affects plants by causing water deficit in plant cells as a result of osmotic effects and inhibition of many enzymes in primary and secondary metabolisms^5^, and this is achieved through (1) reducing the water potential inside plant cells due to the presence of high concentration of ions such as Na^+^ and Cl^−^, (2) causing ionic stress or toxicity by disrupting K^+^/Na^+^ homeostasis and inactivation of many important enzymes in plant cells^6^. Water deficit stress leads to inhibition of root and shoot development due to the reduction in cell division and elongation, alters flowering times and number, and inhibits seed development and production^7^.

It was well known that increased salt tolerance could be achieved by reducing the cytosolic Na^+^ concentrations^8^. Plants can lower the concentration of Na^+^ by excluding Na^+^ via plasma membrane-bound Na^+^/H^+^ antiporter such as SOS1^9^ or sequestration of Na^+^ into vacuole via the tonoplast membrane-bound Na^+^/H^+^ antiporter such as NHX1^10^. The activity of NHX1 depends on the proton gradient across the tonoplast membrane that is created by proton pumps such as H^+^-ATPase and vacuolar H^+^-pyrophosphatase^11^. The Arabidopsis gene *AVP1* encodes a vacuolar H^+^-pyrophosphatase that pumps H^+^ into vacuole and overexpression of *AVP1* leads to increased salt tolerance, which was attributed to Na^+^-sequestration into the vacuolar lumen at the expense of protons^12^. Overexpression of *AVP1* also leads to increased auxin polar transport, thereby resulting in robust root development, leading to higher water absorption capacity and increased drought tolerance^13^. Studies with *AVP1*-overexpressing tomato^14^ and cotton^15^ showed a dramatic increase in root biomass and improved plant survival rate under drought and salt stress conditions. Yang *et al*. revealed that the overexpression of *AVP1* in transgenic plants promotes rhizosphere acidification, root proliferation, and fruit development under phosphate starvation conditions^16,17^. It has also been demonstrated that overexpression of the H^+^-PPase gene from *Thellungiella halophila* in maize could enhance plant response to limiting phosphate concentrations^18^. Lv *et al*. reported a significant improvement in growth and photosynthetic rates of cotton plants overexpressing the H^+^-PPase gene from *Thellungiella halophila* in response to salt stress^19^. In addition, the positive correlation between nitrate use efficiency and *AVP1* up-regulation in lettuce was shown^20^. Several studies on overexpression of *AVP1* or another type I H^+^-PPase genes have shown a substantial improvement in salt and drought tolerance in various plants including cotton^15,19,21^, creeping bentgrass^22^, maize^23^, peanut^24^, sugarcane^25^, tobacco^26^, and rice^27^. Schilling *et al*. showed that overexpression of *AVP1* increased yield and shoot biomass in barely plants grown in saline soil^28^.

Heat stress is another environmental stress that results in significant losses in crop production worldwide^29^, and it is predicted to become even more serious in the future^30^. It has been shown that an increase in air temperature above a plant’s optimum growth temperature decreases net photosynthetic rates in plants^31^ and affects membrane fluidity, which disrupts ion hemostasis in plant cells^32^. Therefore, increasing heat tolerance in crops may allow plants to maintain metabolic function and growth under heat stress conditions. Efforts have been made in engineering plants for heat stress tolerance by overexpressing heat-shock protein genes or heat-shock factor genes in transgenic plants over the last 20 years^3^, yet limited success was made, and so far none was applied in crops.

Recent discoveries might change the landscape of transgenic research on heat-tolerant plants. Overexpression of *OsSIZ1*, a SUMO E3 ligase gene from rice, was found to confer increased heat and drought tolerance in transgenic creeping bentgrass^33^ and transgenic cotton^34^.

SUMO stands for small ubiquitin-like modifier and it can be added to proteins via a post-translational process called sumoylation by a three-enzyme system: SUMO E1 activating enzyme, SUMO E2 conjugating enzyme, and SUMO E3 ligase^35^. The sumoylation reactions carried out by SUMO E3 ligases are important to many regulatory proteins, as sumoylation might alter protein-protein interactions, change protein activities or subcellular localization of proteins^35^. The importance of the Arabidopsis SUMO E3 ligase SIZ1 in plant growth, development, and response to hormones, abiotic and biotic stresses have been studied extensively in Arabidopsis^35^. For examples, and the roles of SIZ1 in sugar signaling^36^, in plant response to drought^37^ and cold temperature stress^38^ were well documented. Other studies also illustrated the function of SIZ1 in regulating phosphate starvation response^39^ and nitrogen assimilation in Arabidopsis through sumoylation of nitrate reductase^40^. Yoo *et al*. showed that *siz1* mutants are thermal hypersensitive, indicating that SIZ1-dependent sumoylation is required for heat tolerance^41^. These studies showed that the Arabidopsis SIZ1 and its homolog in rice, OsSIZ1, are important in the establishment of heat and drought tolerance in plants. Our recent research^34^ indicates that *OsSIZ1-*overexpressing cotton plants performed better than any other transgenic cotton plants that we have created over the last 20 years, which includes *AtNHX1*-overexpressing cotton plants^42^, *AVP1*-overexpressing cotton plants^15^, and *IPT*-transgenic cotton plants^43^.

Given the important role of *AVP1* and *SIZ1* in plant abiotic stress response, we hypothesized that co-overexpression of *AVP1* and *OsSIZ1* would have an additive effect on tolerance to water deficit, salinity, and high-temperature stress in Arabidopsis. Our findings show that *AVP1*/*OsSIZ1* co-overexpression significantly enhanced tolerance to these stresses and serves as a proof-of-concept that co-overexpression of these genes has the potential to improve yields in crop plants significantly.

## Results

### Creation and molecular analysis of *AVP1*/*OsSIZ1* co-overexpressing plants

**Figure 1:**
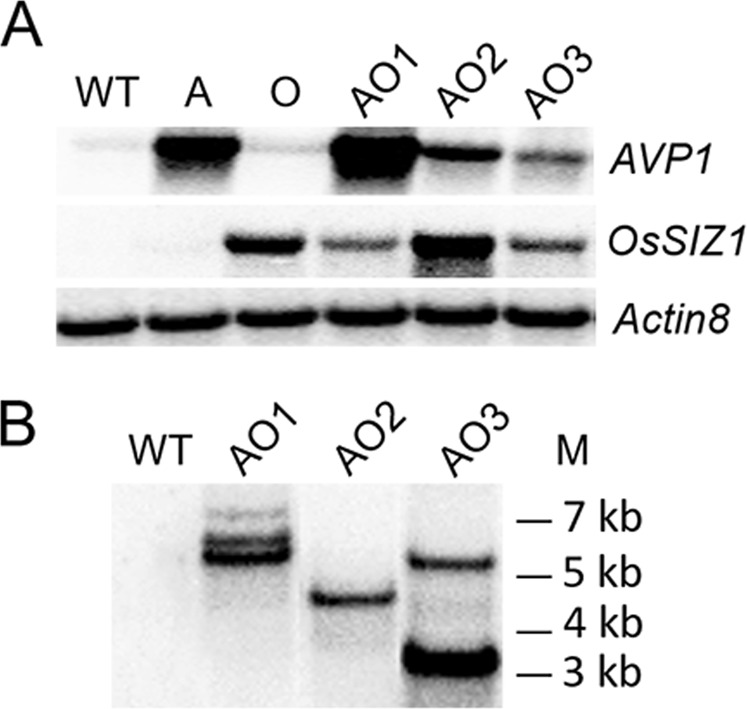
Molecular analysis of wild-type and *AVP1/OsSIZ1* co-overexpressing plants. (**A**) RNA blot analysis of wild-type, *AVP1*-overexpressing, *OsSIZ1*-overexpressing, and *AVP1*/*OsSIZ1* co-overexpressing plants. (**B**) DNA blot analysis of wild-type and *AVP1*/*OsSIZ1* co-overexpressing plants. WT, wild-type plant; A, *AVP1*-overexpressing plant; O, *OsSIZ1*-overexpressing plant; AO1 to AO3, three independent *AVP1*/*OsSIZ1* co-overexpressing plants. M, molecular weight markers.

The expression cassette of the pdual35S-*AVP1*/pUbi-*OsSIZ1* was cloned into a pBI121 based binary vector and introduced into *Arabidopsis thaliana*, using Agrobacterium-mediated “floral dip” transformation^44^. Forty independent transgenic lines were identified by screening on MS media supplemented with 30 µg/ml of kanamycin. DNA was isolated from kanamycin-resistant lines, and the presence of *AVP1* and *OsSIZ1* was verified by polymerase chain reaction (PCR). RNA blot analysis was performed to verify transgene expression, and three independent *AVP1*/*OsSIZ1* co-overexpressing lines, AO1, AO2, and AO3 were selected for further experiments (Fig. 1A). The two reference lines expressing either *AVP1* or *OsSIZ1* were used as positive controls for the RNA blot (Fig. 1A). Also, we conducted DNA blot analysis to confirm the stable integration of transgenes into the Arabidopsis genome and the copy numbers of transgenes in these transgenic plants. Our data indicated that AO1, AO2, and AO3 plants are likely due to three, single, and two T-DNA insertion events, respectively (Fig. 1B).

### *AVP1*/*OsSIZ1* co-overexpression results in enhanced tolerance to salt, heat, and water deficit stresses

**Figure 2:**
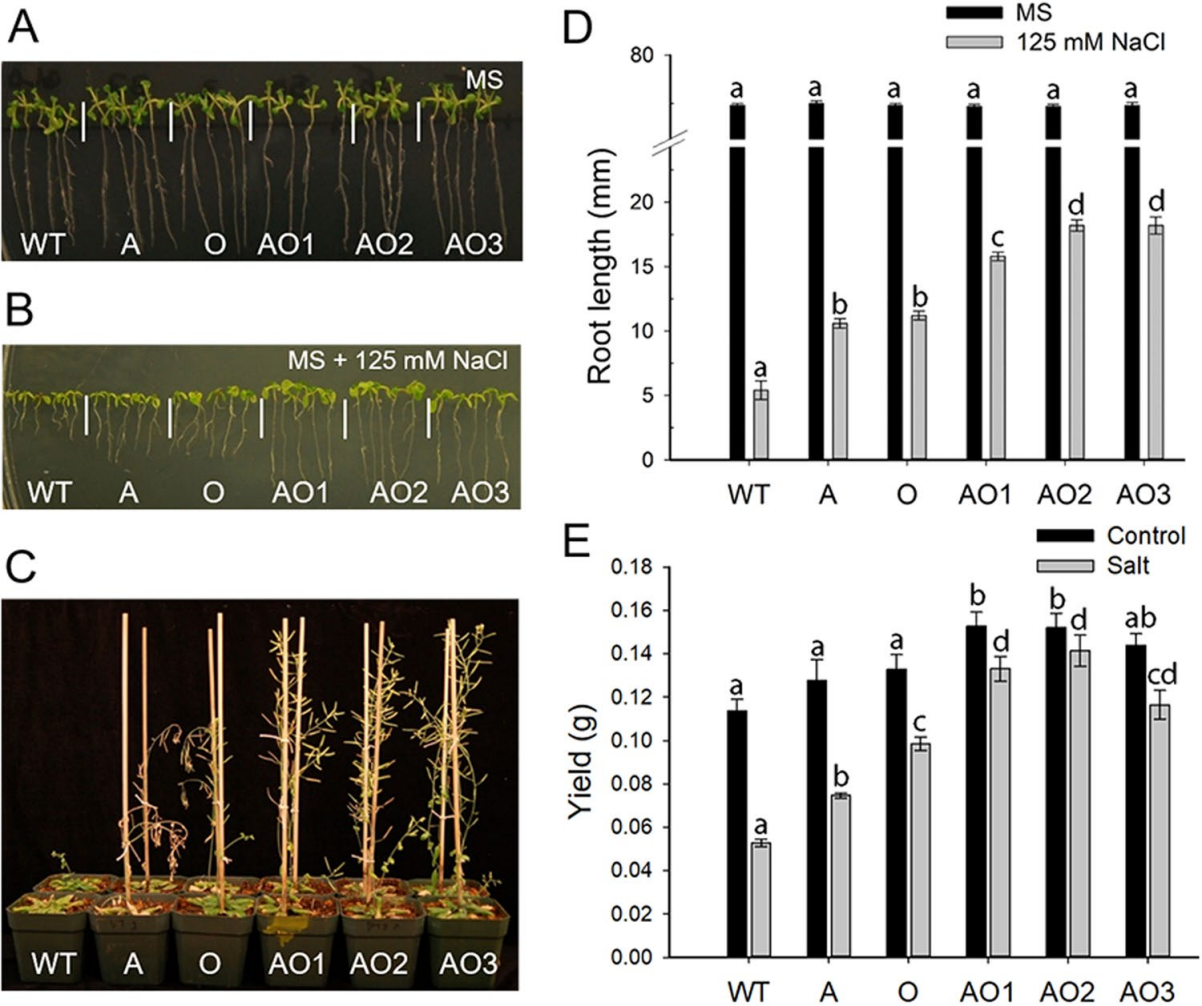
Analysis of wild-type, *AVP1*-overexpressing, *OsSIZ1*-overexpressing, and *AVP1*/*OsSIZ1* co-overexpressing plants under salt stress conditions. (**A**) Phenotypes of wild-type and transgenic plants on MS plate. (**B**) Phenotypes of wild-type and transgenic plants on MS plate supplemented with 125 mM of NaCl. (**C**) Phenotypes of wild-type and transgenic plants under salt stress condition (150 mM of NaCl) in soil. (**D**) Analysis of root length of wild-type and transgenic plants on MS plates shown in A and B above and in Supp. Fig. 2A. (**E**) Seed yields of wild-type and transgenic plants grown in soil under control and salt stress conditions. Total seed yield per plant grown under normal irrigation (black bar) and salt stress (grey bar) conditions were determined at the end of salt stress treatment. Data are means ± SE (n = 9). WT, wild-type plant; A, *AVP1*-overexpressing plant; O, *OsSIZ1*-overexpressing plant; AO1 to AO3, three independent *AVP1*/*OsSIZ1* co-overexpressing plants. Samples denoted by different letters are significantly different (*P* < 0.05, ANOVA, Tukey correction).

A series of experiments were conducted to examine the response of *AVP1/OsSIZ1* co-overexpressing plants (AO), *AVP1*-overexpressing plants (A), *OsSIZ1*-overexpressing plants (O), and wild-type plants (WT) to salt, heat, and water deficit stresses. Plants were grown on MS plates and in potting soil for all experiments and control conditions were defined as PPFD = 175 µmol m^−2^ s^−1^, 16 h light/8 h dark at 22/17 °C day/night temperature regime. Supplementary Fig. 1A–E shows no differences in morphology, growth rate, and flowering time among WT, A, O, and AO plants are grown on MS or in potting soil under control conditions. However, *AVP1/OsSIZ1* co-overexpressing plants consistently produced more siliques and seeds compared to other genotypes under control conditions. Under salt stress conditions, *AVP1/OsSIZ1* co-overexpressing plants were more salt tolerant than *OsSIZ1*-overexpressing and *AVP1*-overexpressing plants, and WT plants were the most sensitive to salt stress treatment (Fig. 2 and Supp. Fig. 2). Under normal growth condition (MS alone), there were no apparent differences in root phenotypes (Fig. 2A), whereas in the presence of 125 mM and 150 mM NaCl, *AVP1*/*OsSIZ1* co-overexpressing plants produced significantly longer roots (Fig. 2B,D, and Supp. Fig. 2A).

For studying soil-grown plants under salt stress treatment, seeds were sown into potting soil and grown for three weeks under control conditions, and salt stress was subsequently applied in the amount of 50 mM in the beginning for four days, then increased to 100 mM for four days and 150 mM for four days. After that, regular water was used for irrigation until the end of the experiment. Our soil experiment results show that AO plants out-performed A, O, and WT plants and produced 150% more seed yield compared to WT under salt stress condition (Fig. 2C,E, and Supp. Fig. 2C,D). We also measured the concentration of Na^+^ and K^+^ ions in leaves of WT and transgenic plants under control and salt stress conditions, and we found that AO plants irrigated with saline water contained around 20% and 30% more Na^+^ and K^+^ ions than WT plants, respectively (Supp. Fig. 3).

**Figure 3:**
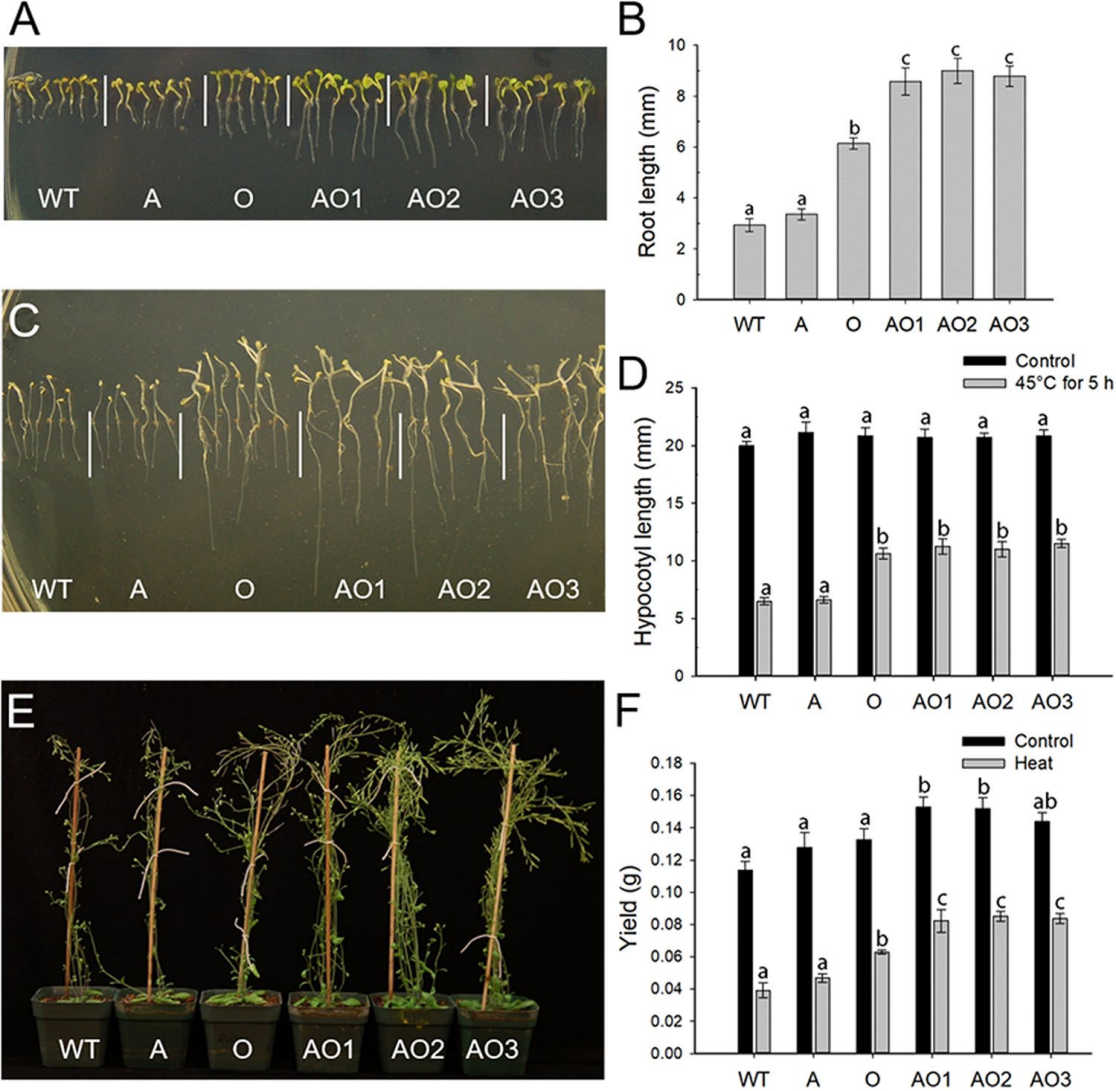
Analysis of wild-type, *AVP1*-overexpressing, *OsSIZ1*-overexpressing, and *AVP1*/*OsSIZ1* co-overexpressing plants under heat stress conditions. (**A**) Phenotypes of wild-type and transgenic plants on MS plate exposed to 32 °C for ten days. (**B**) Root length analysis of plants shown in A. (**C**) Phenotypes of dark-grown plants on MS plate exposed to 45 °C for 5 h. (**D**) Hypocotyl length analysis of dark-grown plants at room temperature (black bar) and exposed to 45 °C for 5 h (grey bar). Data are means ± SE (n = 6). (**E**) Phenotypes of wild-type and transgenic plants grown in soil under heat stress treatment at 37 °C for 5.5 h per day for 40 days. (**F**) Total seed yields of plants grown under normal growth condition (black bar) and heat stress condition (grey bar). Data are means ± SE (n = 9). WT, wild-type plant; A, *AVP1*-overexpressing plant; O, *OsSIZ1*-overexpressing plant; AO1 to AO3, three independent *AVP1*/*OsSIZ1* co-overexpressing plants. Samples denoted by different letters are significantly different (*P* < 0.05, ANOVA, Tukey correction).

Figure 3 shows the response of WT, A, O, and AO to elevated temperature. Figure 3A,B shows the phenotypes of WT and transgenic plants on MS media after moderate heat treatment (i.e., 32 °C) for ten days. *AVP1*/*OsSIZ1* co-overexpressing plants produced significantly longer roots compared to O, A, and WT plants and O had significantly longer roots than A and WT plants. There were no differences in root length between A and WT plants. This was expected as the increased heat tolerance is conferred by overexpression of *OsSIZ1*, not *AVP1*. Interestingly, the significant difference in root length observed in AO plants compared to O plants suggests that there is potentially a synergism between *AVP1*-overexpression and *OsSIZ1*-overexpression (Fig. 3B). Interestingly, this synergy was not observed when the hypocotyl length of dark-grown plants was analyzed. In this assay, 3-day old seedlings grown on MS plates in darkness were exposed to 45 °C for five h in darkness and then moved to room temperature for another ten days in darkness before the hypocotyl lengths of plants were measured (Fig. 3C). The hypocotyl lengths of AO and O plants were similar, but both lines showed longer hypocotyls compared to A and WT plants (Fig. 3D).

The performance of soil-grown plants under heat stress was also examined. Seeds were sown into potting soil and grown for three weeks under control conditions and subsequently transferred to a growth chamber where daytime growth temperature was ramped up to 37 °C in the middle of the day for 5.5 h per day for 40 days. No significant differences in growth rate or plant height were observed (Fig. 3E), but AO and O plants produced more shoot branches and more siliques compared to A and WT plants (Fig. 3E), and consequently the highest seed yields (e.g., 110% more in AO plants than in WT plants (Fig. 3F).

**Figure 4:**
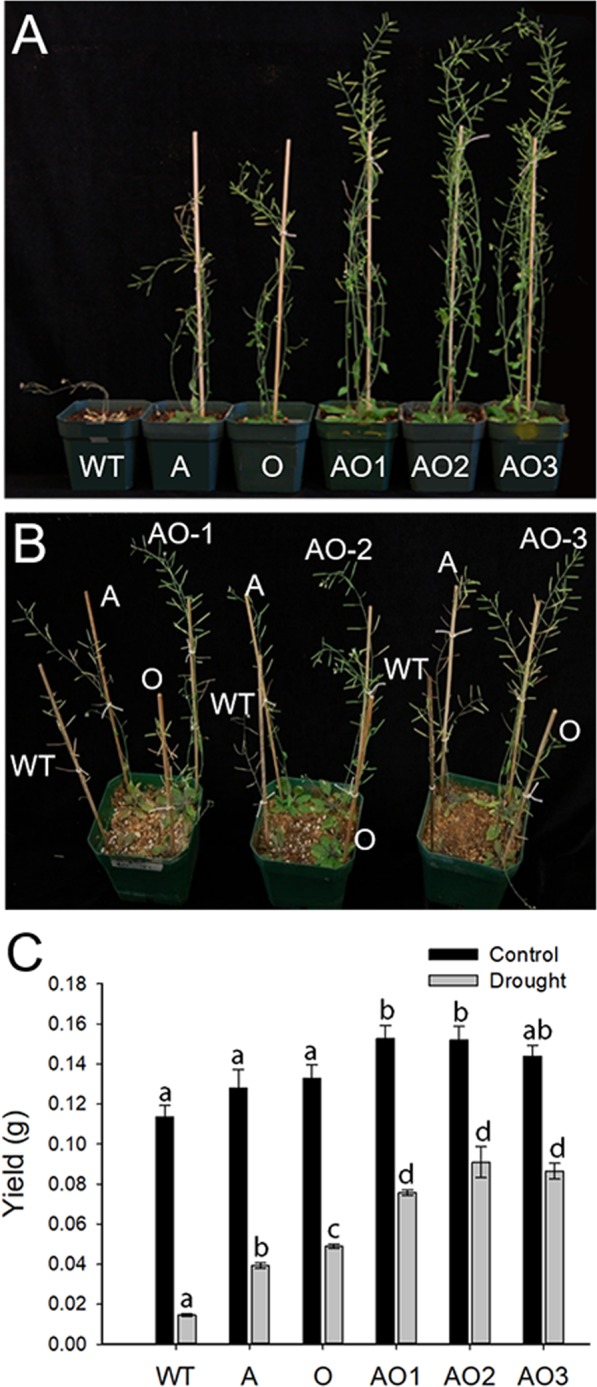
Analysis of wild-type, *AVP1*-overexpressing, *OsSIZ1*-overexpressing, and *AVP1*/*OsSIZ1* co-overexpressing plants under drought stress condition. (**A**) Phenotypes of wild-type and transgenic plants grown under reduced irrigation condition. (**B**) Phenotypes of wild-type and transgenic plants grown in the same pot under reduced irrigation condition. (**C**) Total seed yields of plants grown under normal growth condition (black bar) and reduced irrigation condition (grey bar). Data are means ± SE (n = 9). WT, wild-type plant; A, *AVP1*-overexpressing plant; O, *OsSIZ1*-overexpressing plant; AO1 to AO3, three independent *AVP1*/*OsSIZ1* co-overexpressing plants. Samples denoted by different letters are significantly different (*P* < 0.05, ANOVA, Tukey correction).

Figure 4 shows the response of WT, A, O, and AO plants to water deficit stress. Figure 4A,B shows the phenotype of 7-week old plants after exposure to 2 water deficit stress events. Briefly, plants were grown under control conditions for three weeks followed by a slow-onset water deficit stress for two weeks, irrigation to field capacity; then the second water deficit stress was applied for two weeks. Figure 4A,B shows that *AVP1/OsSIZ1* co-overexpressing plants outperformed A, O, and WT plants. Under water deficit stress conditions, AO plants were larger than the other genotypes and had more siliques (Supp. Fig. 4) and showed a 2-fold increase in seed production compared to A and O plants and over 4-fold increase in seed production compared to WT plants (Fig. 4C).

### *AVP1*/*OsSIZ1* co-overexpression results in enhanced tolerance to simultaneous abiotic stress events

**Figure 5:**
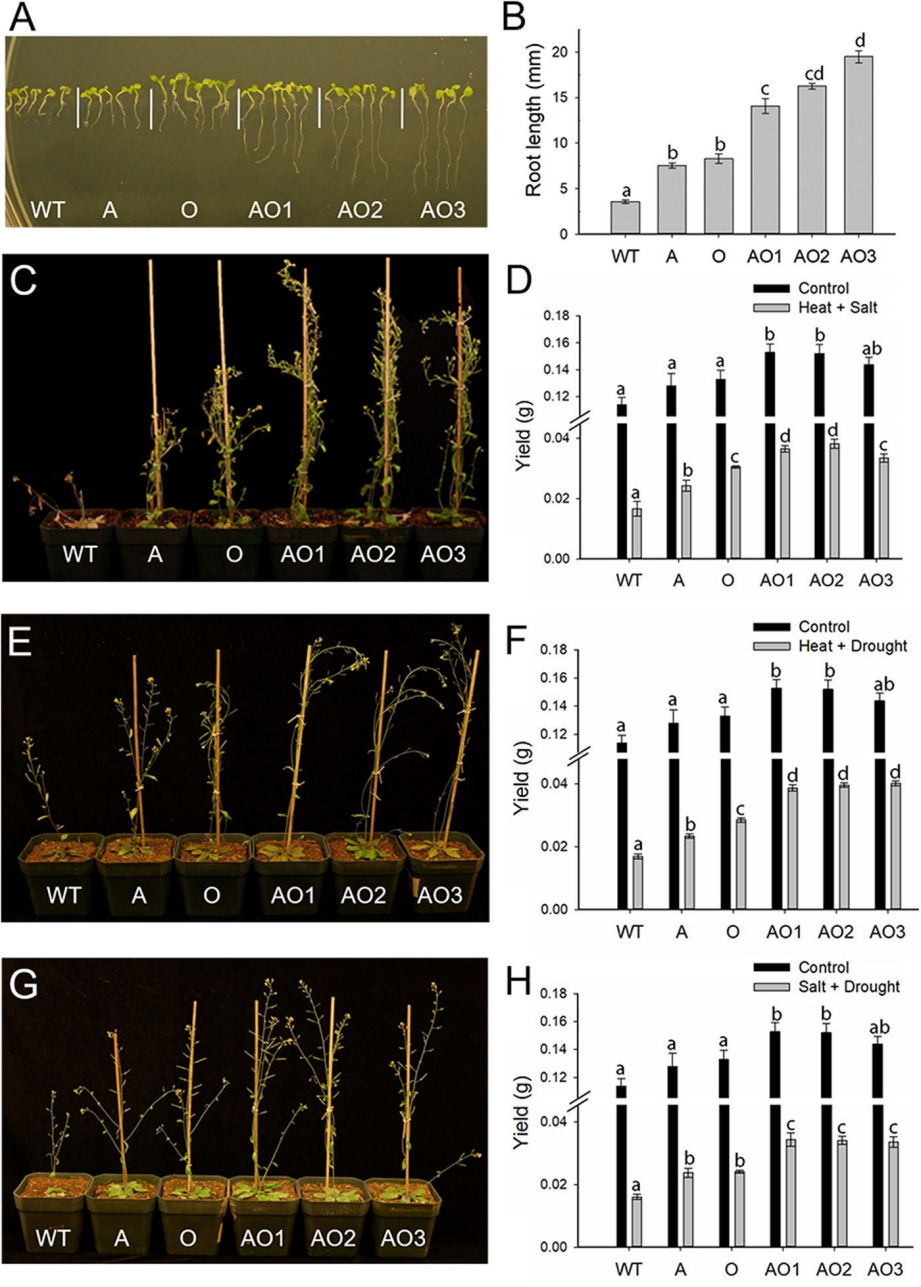
Analysis of wild-type, *AVP1*-overexpressing, *OsSIZ1*-overexpressing, and *AVP1*/*OsSIZ1* co-overexpressing plants under multi-stress conditions. (**A**) Phenotypes of wild-type and transgenic plants grown under combined heat (32 °C) and salt (100 mM NaCl) stresses for ten days. (**B**) Root length analysis of plants grown under combined heat and salt stresses as shown in A. (**C**) Phenotypes of wild-type and transgenic plants grown under combined heat and salt stresses in soil. (**D**) Total seed yields of plants grown under normal condition (black bar) and combined heat and salt stresses (grey bar). Data are means ± SE (n = 9). (**E**) Phenotypes of wild-type and transgenic plants grown under combined heat and drought stresses in soil. (**F**) Total seed yields of plants grown under normal growth condition (black bar) and combined heat and drought stresses (grey bar). Data are means ± SE (n = 9). (**G**) Phenotypes of wild-type and transgenic plants grown under combined salt and drought stresses in soil. (**H**) Total seed yields of plants grown under normal condition (black bar) and combined salt and drought stresses (grey bar). Data are means ± SE (n = 9). WT, wild-type plant; A, *AVP1*-overexpressing plant; O, *OsSIZ1*-overexpressing plant; AO1 to AO3, three independent *AVP1*/*OsSIZ1* co-overexpressing plants. Samples denoted by different letters are significantly different (*P* < 0.05, ANOVA, Tukey correction).

We also examined the response of WT, A, O, and AO plants to multiple, simultaneous stress events. Figure 5 shows the growth and seed yield response to multiple stress conditions. Root growth was enhanced in all transgenic lines compared to WT plants grown on MS + 100 mM NaCl and the continuous growth temperature of 32 °C, with *AVP1*/*OsSIZ1* co-overexpressing plants showing over 2-fold increase over A and O and an almost 4-fold increase in root length compared to WT (Fig. 5A,B). Similarly, for soil-grown plants, all transgenic lines produced more above-ground biomass and more seed yield compared to WT plants when grown under heat + salt (Fig. 5C,D and Supp. Fig. 5), heat + water deficit (Fig. 5E,F and Supp. Fig. 6), and salt + water deficit (Fig. 5G,H and Supp. Fig. 7). In all conditions, *AVP1*/*OsSIZ1* co-overexpressing plants produced significantly more biomass and seeds than all genotypes tested (Fig. 5C–H).

**Figure 6:**
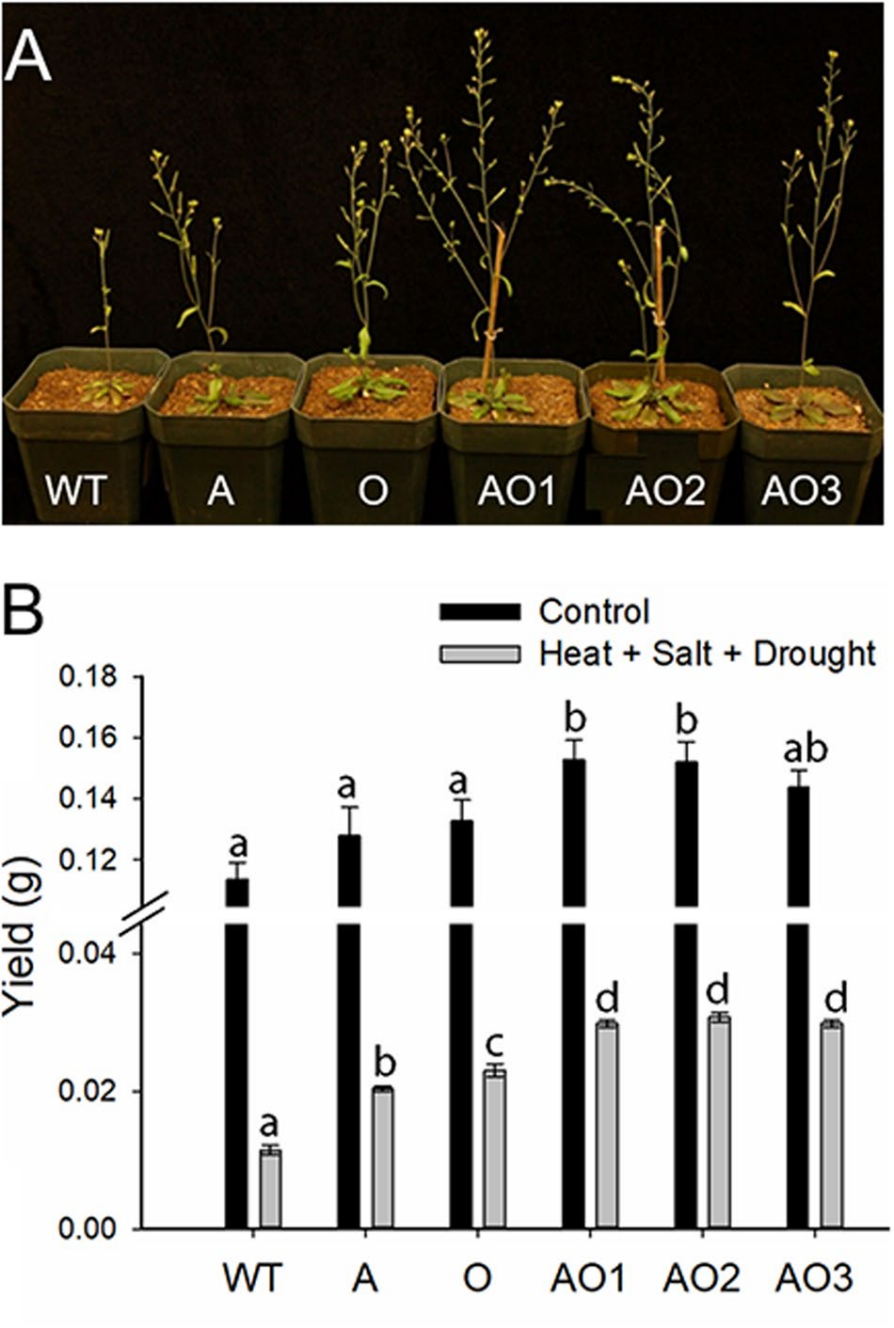
Analysis of wild-type, *AVP1*-overexpressing, *OsSIZ1*-overexpressing, and *AVP1*/*OsSIZ1* co-overexpressing plants under combined drought, heat, and salt stresses. (**A**) Phenotypes of wild-type and transgenic plants grown under combined heat, salt, and drought stresses in soil. (**B**) Total seed yields of plants grown under normal growth condition (black bar) and combined heat, salt, and drought stresses (grey bar). Data are means ± SE (n = 9). WT, wild-type plant; A, *AVP1*-overexpressing plant; O, *OsSIZ1*-overexpressing plant; AO1 to AO3, three independent *AVP1*/*OsSIZ1* co-overexpressing plants. Samples denoted by different letters are significantly different (*P* < 0.05, ANOVA, Tukey correction).

Figure 6 shows the response of WT, A, O, and AO plants to water deficit, salt, and heat stresses (i.e., three-week-old plants were treated with limited irrigation of saline solutions, plus 37 °C for 5.5 h per day until the end of the experiment). Despite severe reductions in total seed yields in all plants due to the harsh conditions created by simultaneous exposure to three stresses, *AVP1*/*OsSIZ1* co-overexpressing plants demonstrated the healthiest phenotype, followed by *AVP1-*overexpressing and *OsSIZ1*-overexpressing plants, and WT plants were the worst (Fig. 6A). These differences in growth and appearance were reflected in seed yield with *AVP1*/*OsSIZ1* co-overexpressing plants producing over 160% more seeds than WT, and 31% and 48% more seeds than *OsSIZ1*-overexpressing and *AVP1*-overexpressing plants, respectively (Fig. 6B).

### *AVP1*/*OsSIZ1* co-overexpressing plants release more protons into media

**Figure 7:**
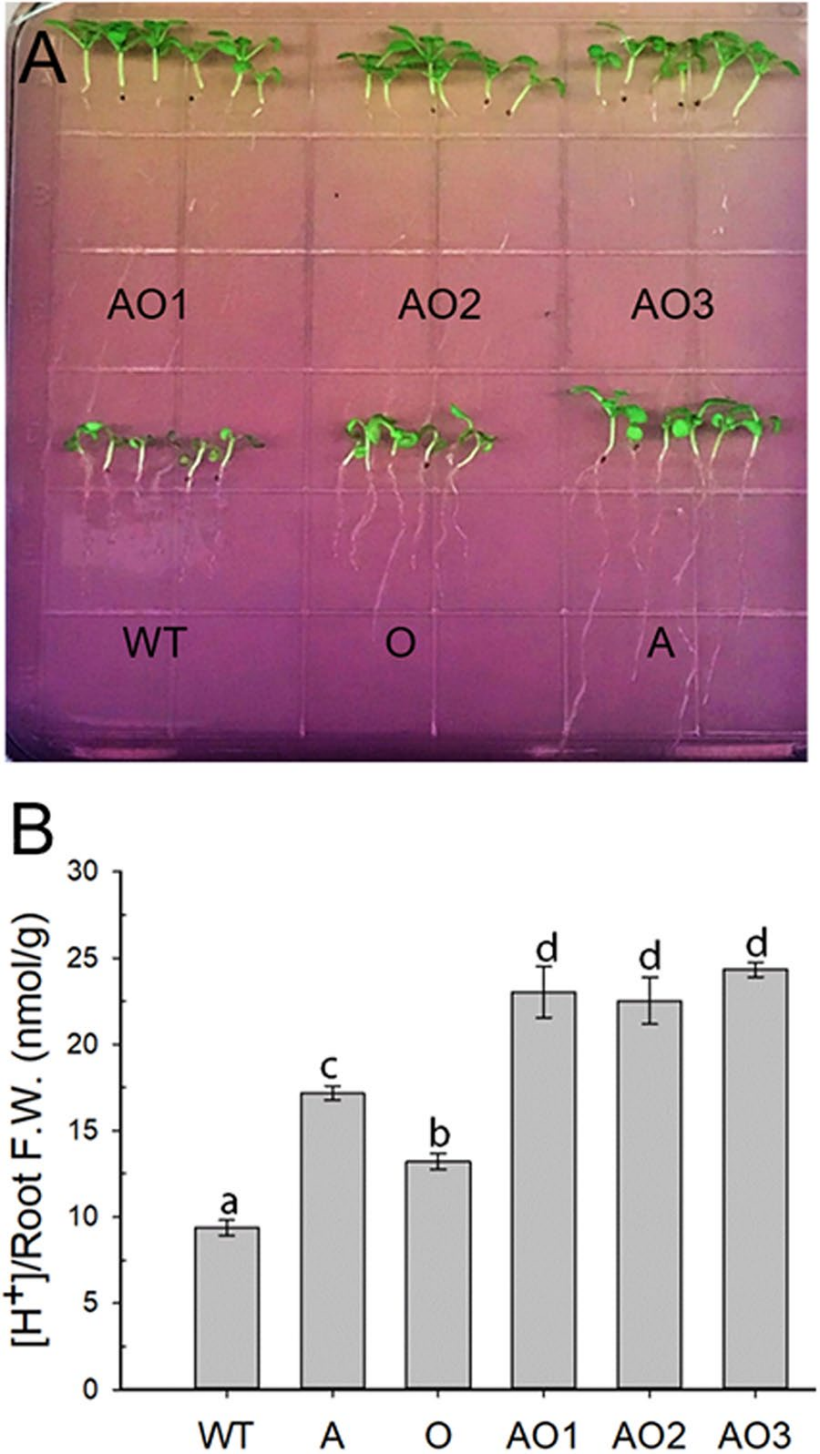
Analysis of root acidification in wild-type, *AVP1*-overexpressing, *OsSIZ1*-overexpressing, and *AVP1*/*OsSIZ1* co-overexpressing plants. (**A**) Phenotype of seedlings of wild-type and transgenic plants grown on MS plate containing the pH indicator bromocresol purple. The color change from purple to yellow indicates the pH change in the media due to acidification of rhizosphere of plants. (**B**) Protons released from the roots of wild-type and transgenic plants. Data are means ± SE (n = 5 pools of 10 plants for each pool). WT, wild-type plant; A, *AVP1*-overexpressing plant; O, *OsSIZ1*-overexpressing plant; AO1 to AO3, three independent *AVP1*/*OsSIZ1* co-overexpressing plants. Samples denoted by different letters are significantly different (*P* < 0.05, ANOVA, Tukey correction).

Since *AVP1*/*OsSIZ1* co-overexpressing plants displayed better phenotypes under all stress conditions tested, the acidification capacity of these plants might be better than other plants as this should be the property of *AVP1*-overexpression^13^. A recent study also showed that the SUMO E3 ligase MdSIZ1 from apple targets MdbHLH104, which modulates the rhizosphere acidification via up-regulation of plasma membrane (PM) H^+^-ATPase^45^. We therefore conducted the acidification assay and found that *AVP1*/*OsSIZ1* co-overexpressing plants did have the biggest capacity in acidifying apoplast (see the yellow color in Fig. 7A). *AVP1*/*OsSIZ1* co-overexpressing plants released around 35% more protons into media than *AVP1*-overexpressing plants. *OsSIZ1*-overexpressing plants also released more protons into media than WT plants, which might be due to sumoylation of bHLH transcription factor that results in higher PM H^+^-ATPass-mediated rhizosphere acidification^45^. As data showed that *AVP1*/*OsSIZ1* co-overexpressing plants have around two-fold increase in the ability to acidify the environment as compared to wild-type plants.

### Co-overexpression of *AVP1*/*OsSIZ1* significantly alters gene expression under abiotic stresses

**Figure 8:**
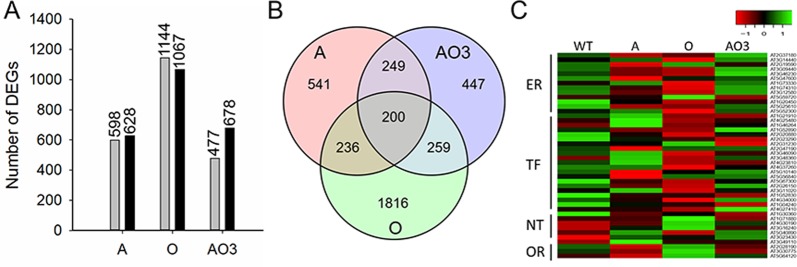
RNA sequencing analysis of wild-type, *AVP1*-overexpressing, *OsSIZ1*-overexpressing, and *AVP1*/*OsSIZ1* co-overexpressing plants under combined heat and drought stresses. (**A**) Number of differentially expressed genes in transgenic plants under combined heat and drought stresses. Grey bars represent down-regulated genes, and black bars represent up-regulated genes. (**B**) Venn diagram of differentially expressed genes in transgenic plants vs. wild-type plants under combined heat and drought stresses. (**C**) Heatmap of 44 stress-related differentially expressed genes. WT, wild-type plant; A, *AVP1*-overexpressing plant; O, *OsSIZ1*-overexpressing plant; AO3, *AVP1*/*OsSIZ1* co-overexpressing plants. DEGs, differentially expressed genes; ER, environmental response; TF, transcription factor; NT, nutrient transfer; OR, oxidation-reduction.

To understand the molecular basis of the substantially improved abiotic stress tolerance in *AVP1*/*OsSIZ1* co-overexpressing plants, RNA transcript profiles were generated for WT, A, O, and AO leaf tissues exposed to simultaneous heat and water-deficit stresses. The RNA sequencing data showed that there are 1155 differentially expressed genes (DEGs) in *AVP1*/*OsSIZ1* co-overexpressing plant (AO3) compared to WT plants (Fig. 8A). Among 1226 DEGs in *AVP1*-overexpressing plants and 2211 DEGs in *OsSIZ1*-overexpressing plants under combined heat and drought stresses, 628 and 1067 were up-regulated, and 598 and 1144 were down-regulated in *AVP1*-overexpressing and *OsSIZ1*-overexpressing plants, respectively (Fig. 8A). The numbers of DEGs in *AVP1*-overexpressing, *OsSIZ1*-overexpressing, and *AVP1*/*OsSIZ1* co-overexpressing (AO3) plants in comparing to those of WT plants under normal growth condition are presented in Supp. Fig. 8A. The Venn diagrams illustrated in Fig. 8B and Supp. Fig. 8B provide an overview of the distribution of DEGs in transgenic plants in comparing to those of WT plants under combined heat and drought stresses and normal growth conditions. According to these data, 449 and 459 DEGs in AO3 plants vs. WT plants share similar patterns with *AVP1*-overexpressing and *OsSIZ1*-overexpressing plants, respectively, while *AVP1*-overexpressing plants and *OsSIZ1*-overexpressing plants shared 436 DEGs under combined heat and drought stresses. A total of 200 DEGs were up- or down-regulated in *AVP1*-overexpressing, *OsSIZ1*-overexpressing, and *AVP1*/*OsSIZ1* co-overexpressing plants under combined heat and drought stress conditions (Fig. 8B). Under normal growth condition (i.e., optimum temperature and regular irrigation), AO3 shared 428 and 403 DEGs with *AVP1*-overexpressing plants and *OsSIZ1*-overexpressing plant, respectively, and 142 DEGs were shared in all three plants (Supp. Fig. 8B).

Among those shared 200 DEGs, we selected 44 DEGs (Supp. Table 1) with the fold-of-change >2, including 21 transcription factor (TF) genes, 13 environmental response (ER) associated genes, 6 nutrient transfer (NT) genes, and 4 oxidative stress response (OR) genes for further analysis (Fig. 8C). Under combined heat and water-deficit stresses, heat shock protein (HSP) genes (e.g., *HSP70* and *HSP101*) were up-regulated in AO3 plant, with significantly higher levels of transcripts (log_2_-fold >7.5) (Supp. Table 1). A member of small heat shock proteins (sHSPs), HSP17.4, was significantly up-regulated in AO3 plant (log_2_-fold of 10.3) in comparing to that of WT plants (log_2_-fold of 8.8). These data are consistent with the functions of HSPs as chaperones in stabilizing proteins and re-folding damaged proteins under stress condition^46^. The transcript levels of several TF genes (i.e., *NAC19*, *HSFA2*, and *ABF3*) were also altered. Since many TFs are involved in regulating the expression of stress-related genes, any changes in the expression pattern of TF genes will likely result in higher stress tolerance in transgenic plants. The RNA sequencing results also exhibited that, the gene *RD28* was highly expressed (log_2_-fold > 3.6) in AO3 plants in comparing to that in WT plants under combined heat and drought stresses, however, the transcript of *ERD4* was slightly downregulated in AO3 plant. Notably, the transcript of *NCED3* was much higher in AO3 plants than those of WT, *AVP1*-overexpressing, and *OsSIZ1*-overexpressing plants under combined heat and drought stresses, indicating that ABA-dependent pathway might be involved in the improved drought tolerance in *AVP1/OsSOZ1* co-overexpressing plant^47^.

### Quantitative real time-PCR analysis confirmed RNA sequencing results

**Figure 9:**
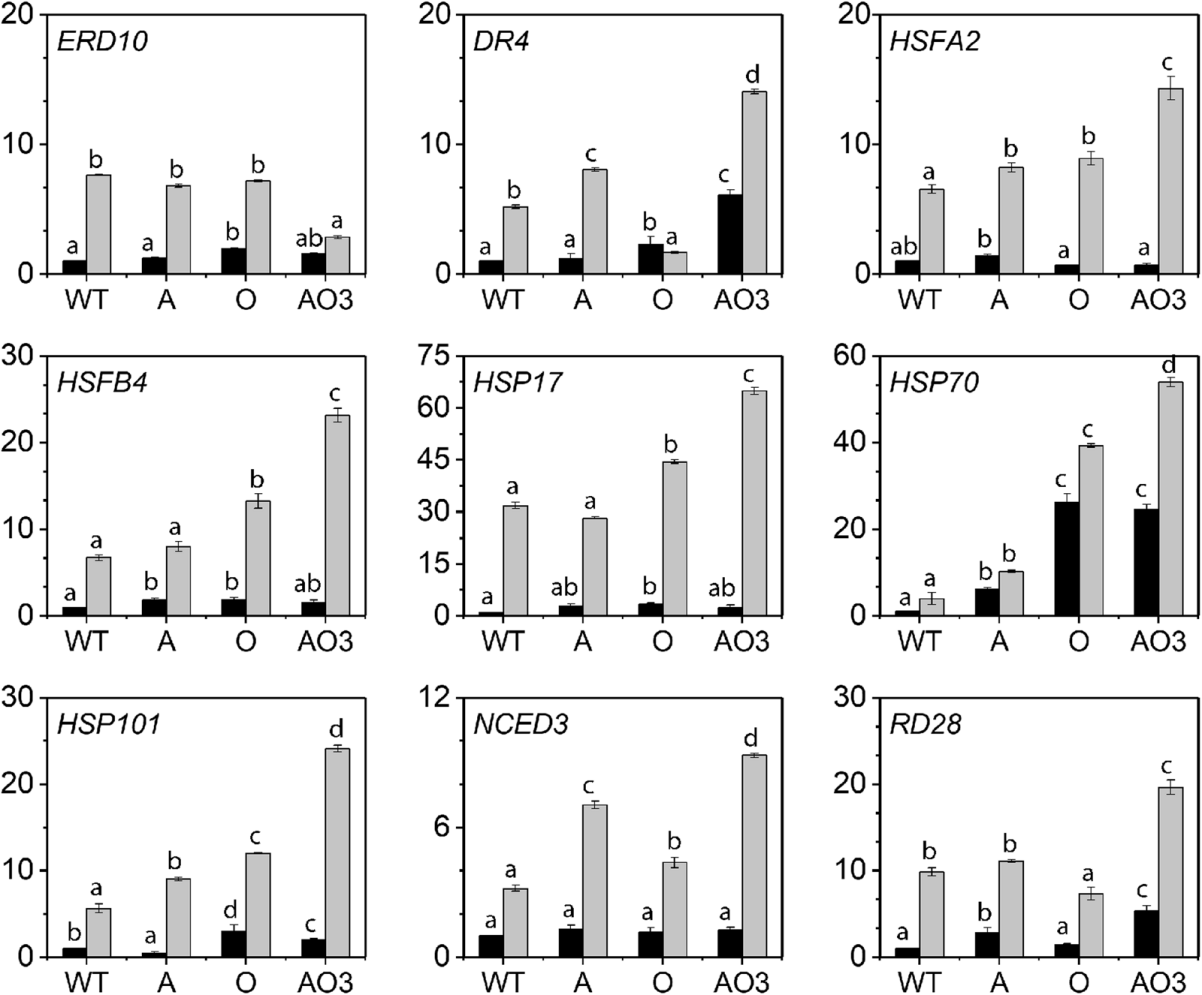
Quantitative real-time PCR (qRT-PCR) analysis of nine stress-related genes in wild-type, *AVP1*-overexpressing, *OsSIZ1*-overexpressing, and *AVP1*/*OsSIZ1* co-overexpressing plants under normal growth condition (black bar) and combined heat and drought stresses (grey bar). The *Arabidopsis* gene *Actin8* was used as the internal reference gene. The relative transcript level was calculated using the 2^−ΔΔCt^ method. Data are means ± SE (n = 3). WT, wild-type plant; A, *AVP1*-overexpressing plant; O, *OsSIZ1*-overexpressing plant; AO3, *AVP1*/*OsSIZ1* co-overexpressing plants. Samples denoted by different letters are significantly different (*P* < 0.05, ANOVA, Tukey correction).

To confirm the RNA sequencing data, we randomly selected nine genes and conducted quantitative real-time (qRT)-PCR using the gene-specific primers. The *Actin 8* was used as the internal reference gene. The results of the qRT-PCR experiments showed a highly correlated pattern of transcript levels with the RNA sequencing data (Fig. 9). It appears that the transcript levels of most selected genes were already at much higher levels, which might enable transgenic plants to deal with abiotic stresses more effectively in the very beginning, consequently reducing damages caused by the stresses. Other than the heat shock factor genes and small HSP genes (e.g., *HSFA2* and *HSP17*), most transcripts remain at levels higher than that of WT plants (Fig. 9).

## Discussion

Since water deficit, heat, and salinity often occur simultaneously, both in nature and crop production settings in arid and semi-arid regions of the world, creating germplasm with enhanced tolerance to these stresses is of great value. In this study, we demonstrate that co-overexpression of the Arabidopsis vacuolar H^+^-pyrophosphatase gene *AVP1* and the rice SUMO E3 ligase gene *OsSIZ1* significantly enhances plant tolerance to water deficit, heat, and salt stress in transgenic Arabidopsis plants. The increased tolerances to abiotic stresses are significantly higher than those achieved by overexpression of *AVP1* or *OsSIZ1* alone, confirming our hypothesis that co-overexpression of *AVP1* and *OsSIZ1* would further increase tolerance to abiotic stresses, which provides a proof-of-concept that might be applicable to crops, which could potentially lead to substantially increased crop yields in the future.

Previously, Gaxiola *et al*. showed increased tolerance to drought and salt by overexpressing *AVP1* in transgenic Arabidopsis and tomato plants, which was attributed to increased sequestration and accumulation of Na^+^ in vacuoles and increased auxin polar transport^12–14^, thereby leading to robust root development. The sequestration of more Na^+^ into the vacuole is likely due to increased activity of Na^+^/H^+^ antiporters on tonoplast membranes in response to higher H^+^ gradient in vacuole in *AVP1*-overexpressing plants^48^. Overexpression of *OsSIZ1* in transgenic bentgrass and cotton led to increased heat tolerance and drought tolerance in transgenic plants^33,34^. Furthermore, sumoylation appears to help plants to withstand stress conditions via several pathways like protecting the electron transport system, which reduces the negative impacts of stress on photosynthesis^49^. When *AVP1* and *OsSIZ1* were co-overexpressed in Arabidopsis, transgenic plants performed significantly better than *AVP1*-overexpressing plants and *OsSIZ1*-overexpressing plants under all stress conditions tested. Our data indicate that *AVP1*/*OsSIZ1* co-overexpressing plants outperformed other plants under salt stress (Fig. 2), heat stress (Fig. 3), drought stress (Fig. 4), combined heat and salt (Fig. 5C,D), combined heat and drought (Fig. 5E,F), combined drought and salt (Fig. 5G,H), and combined heat, drought and salt (Fig. 6). The improved seed yields under these abiotic stress conditions might be due to simple addition of increased beneficial traits of overexpression of *AVP1* and *OsSIZ1*, and the improved abiotic stress tolerance is significantly better than what has been achieved in prior engineering efforts for abiotic stress tolerance, therefore, this discovery has a far-reaching implication in genetic engineering crops for yield improvement. Our previous work on transgenic cotton plants showed that *AVP1*-overexpression routinely resulted in approximately 20% higher fiber yields than WT cotton when grown under rainfed conditions^15^. Similarly, *OsSIZ1*-overexpressing cotton routinely produced 25% higher fiber yields than WT cotton under rainfed conditions^34^. Based on the research reported here, we predict that *AVP1*/*OsSIZ1* co-overexpressing cotton would produce fiber yields that could exceed the single gene overexpressing cotton under rainfed and low input production scenarios and represents an opportunity to stabilize yields in these highly variable and rapidly expanding production regions.

Since salinity imposes water deficit in plant cells and inhibits many important enzymes in plant cellular metabolisms, it is detrimental to plants. Furthermore, when salinity is combined with drought and/or heat, it becomes more dangerous to plant growth and development^4,50^. Our *AVP1*/*OsSIZ1* co-overexpressing plants display significantly increased tolerance to multiple stresses, which would allow us to employ this technology to reduce crop losses caused by environmental stresses in the future. In addition to salt and drought tolerances, *AVP1-*overexpressing plants showed an increased root acidification^13^. *AVP1*-overexpressing plants were shown to have increased H^+^ extrusion from root cells as a function of enhanced H^+^-ATPase pumps on plasma membrane^51^. This provides a great advantage to plants in uptaking nutrients like phosphate, particularly if grown under phosphate deprived condition^16^. The amount of protons released by *AVP1*-overexpressing plants during the day was found to be approximately 40% higher than that of WT plants^51^, which results in higher sugar metabolism and ATP synthesis in those transgenic plants. The study by Zhou *et al*.^45^ shows that overexpression of *MdSIZ1* in Arabidopsis causes in improved growth and chlorophyll production as well as enhanced proton release to medium compared to WT, indicating that SUMO E3 ligase is involved in the up-regulation of PM H^+^-ATPase. In agreement with these studies, we found that *AVP1*/*OsSIZ1* co-overexpressing plants even have higher capacity in acidifying the rhizosphere (Fig. 7A), hinting at the possibility that the improved growth and yield could be related to better absorption of nutrients, increased photo-assimilate production and sucrose mobility in *AVP1*/*OsSIZ1* co-overexpressing plants.

To better understand the underlying molecular mechanism of substantially increased stress tolerance in *AVP1/OsSIZ1* co-overexpressing plants, we conducted a transcriptome analysis of *AVP1/OsSIZ1* co-overexpressing plants and non-transgenic plants grown under normal growth and combined heat and drought stress conditions. As expected, transcript levels for HSP genes, i.e., *HSP70*, *HSP101*, and *HSP17*, are higher in *AVP1/OsSIZ1* co-overexpressing plants under combined drought and heat stresses (Fig. 8). Heat shock transcription factors (HSFs) control the expression of HSPs^52^. Our RNA sequencing data showed that the transcripts of *HSFA2* and *HSFB4* were at higher levels in *AVP1/OsSIZ1* co-overexpressing plants in comparing to WT plants under normal growth conditions. It should be noted that the transcript levels of a large number of transcription factor genes were altered in *AVP1/OsSIZ1* co-overexpressing plants under combined heat and drought stress conditions. Previous studies illustrated a different pattern of overlap among transcripts of abiotic stress response related genes^53,54^. In our study, however, we found a considerable overlap between the transcripts altered in *AVP*-overexpressing, *OsSIZ1*-overexpressing, and *AVP1/OsSIZ1* co-overexpressing plants under combined heat and drought stress conditions (Fig. 8B). Plants exposed to water deprivation accumulate a variety of osmoprotectants such as proline and glycine-betaine^55^. As it was previously reported by Peng *et al*.^56^, the transcript level of the proline dehydrogenase 1 gene (*POX1*), was down-regulated under drought stress condition and the decrease was more significant in *AVP1*-overexpressing plants in comparing to that of WT plants.

Transcripts of several drought stress-related genes were also altered in plants exposed to combined heat and drought stresses. Among those, the transcript levels of *RD28* and *DR4* were at higher levels in *AVP1/OsSIZ1* co-overexpressing plants in comparing with those of WT plants, while the transcript of *ERD10* was at a lower level in *AVP1*-overexpressing and *AVP1/OsSIZ1* co-overexpressing plants. Previous studies in peanut showed that the transcript level of *NCED1* that encodes the 9-cis-epoxycarotenoid dioxygenase 1 was increased under drought condition^57^. We found that the transcript level of *NCED3* was higher in *AVP1*-overexpressing plants, suggesting that ABA might play a role in the higher stress tolerance of *AVP1/OsSIZ1* co-overexpressing plants under combined heat and drought stress conditions.

The results obtained in RNA sequencing analysis were validated by qRT-PCR analysis (Fig. 9), and we found a high correlation in the transcript levels of different genes between qRT-PCR and RNA sequencing studies. It is clear that further analysis of *AVP1*/*OsSIZ1* co-overexpressing plants at gene expression and metabolic levels using transcriptome profiling and bioinformatics tools will continue to give us hints at the molecular mechanism of the immensely improved abiotic stress tolerance observed in *AVP1*/*OsSIZ1* co-overexpressing plants.

## Conclusion

The detrimental effects of combined abiotic stresses on agricultural production demonstrate the need to create crops that can withstand multiple stresses. In this study, we show that co-overexpression of *AVP1* and *OsSIZ1* in Arabidopsis plants could dramatically improve plant performance under multiple stress conditions, especially under combined heat, drought, and salt stresses. If this result could be replicated in staple crops such as rice, maize, wheat, soybeans, significantly increased yields would be expected for rainfed and low-input agricultural production systems, which may lead to another green revolution, stabilizing regional agriculture-based economies, and keeping millions of people fed and clothed.

## Materials and Methods

### Plasmid construction and arabidopsis transformation

The p35S::AVP1/pUbi::OsSIZ1 construct harboring *NptII* (kanamycin resistance gene) was cloned into the pBI121 based binary vector, and introduced into Arabidopsis (ecotype Columbia) using *Agrobacterium*-mediated floral dip method^44^. The rice SUMO E3 ligase gene *OsSIZ1* and the Arabidopsis vacuolar pyrophosphatase gene *AVP1* were under the control of the dual 35S promoter and the maize ubiquitin gene promoter, respectively. Transgenic plants were selected by screening seeds on Murashige and Skoog^58^ (MS) media supplemented with 30 µg/ml of kanamycin. A total of 40 independent putative transgenic plants were obtained on kanamycin plates and were verified by using PCR with *AVP1* and *OsSIZ1* gene-specific primers. Homozygous transgenic plants were obtained at T_3_ generation and then used for all experiments.

### Plant materials and growth conditions

Wild-type Arabidopsis plants (WT), and two reference lines, *OsSIZ1*-overexpressing plant^59^ (O), *AVP1*-overexpressing plant (A) (generated in our lab), and three independent *AVP1*/*OsSIZ1* co-overexpressing plants (AO1 to AO3) were used in all experiments. Plants were grown in LC1 mix in a growth chamber that was set at 21 °C with a photoperiod of 16 h light/8 h dark. For experiments conducted on MS plates, seeds were surface sterilized using 70% (v/v) ethanol and 15% (v/v) commercial bleach followed by rinsing with sterile distilled water four times, then stratified at 4 °C for three days before sowing seeds on MS plates with or without salts. For experiments conducted in soil, seed collectors were used to avoiding seed loss during harvesting, and all seed yield data were the amount of seeds produced per plant.

### RNA and DNA blot analyses

Total RNAs were extracted from leaves of WT and transgenic plants using the TRIzol reagent (Invitrogen, Carlsbad, CA). Ten µg of total RNAs from WT and transgenic plants were separated on 1.2% (W/V) agarose gel and transferred onto BioTrans (+) ^TM^ nylon membranes (Ambion, USA). The RNA blot analysis was carried out by hybridizing the membrane with P^32^-labelled gene-specific probes for *OsSIZ1*, *AVP1*, and *Actin8*, respectively, under the condition as described by Church and Gilbert^60^. A full-length cDNA for *AVP1* was amplified by PCR using the forward and reverse primers of AVP1_F and AVP1_R, and an *Actin8* cDNA was amplified using forward and reverse primers of Actin8_F and Actin8_R, respectively. An *OsSIZ1* cDNA fragment was amplified as described by Mishra *et al*.^34,59^ The PCR amplified DNA fragments were used to make P^32^-labelled gene-specific probes, using the DECAprime^TM^ II DNA Labeling Kit (Life Technology, NY, USA).

Genomic DNAs were isolated from Arabidopsis leaves using the cetyltrimethylammonium bromide method with minor modifications^61^. Fifteen micrograms of genomic DNAs from WT and transgenic plants were digested overnight with *Hind* III (NEB, USA), then the digested DNAs were run on 0.8% agarose gel followed by transferring it onto a BioTrans (+) ^TM^ nylon membrane. The DNA blotting hybridization was performed as previously described by Hu *et al*.^62^

The oligonucleotide primers are listed below:

AVP1_F: ATGGGCGAGCTCGGTACC

AVP1_R: GAGAGACTGGTGATTTGCGGAC

Actin8_F: TCACCACAACAGCAGAGCGGG

Actin8_R: GGACCTGCCTCATCATACTCGG

### Salt stress treatment

Salt stress treatments were conducted on MS plate and in soil, respectively. Arabidopsis seeds of WT and transgenic plants were surfaced sterilized in 15% bleach once, 70% ethanol once, then rinsed with sterile water four times, then sown on MS plates and kept at 4 °C for three days for stratification. These seeds were allowed to germinate vertically for three days before they were transferred to another MS plate supplemented with 150 mM of NaCl to grow for seven more days, and the number of green cotyledons was counted, and the root lengths were measured. In a different experiment, seeds were directly plated on MS plates supplemented with 125 mM of NaCl. Ten days later the root length and the number of lateral roots were measured. WT plants were grown on MS plates for ten days under the same condition as treated plants. For salt stress treatment in soil, plants were allowed to grow under normal growth condition for three weeks, then saline solution of 50 mM of NaCl was used to irrigate plants (twice in 4 days), then the salt concentration was incrementally increased to 100 mM and 150 mM (each concentration twice, and 4 days at each concentration). After that, plants were irrigated with regular water until the harvest. Seed yields, silique numbers, and shoot heights were measured after salt treatment. Control plants were grown separately in the same growth chamber and irrigated with regular water. For each experiment, three biological replicates for genotype were used, and all experiments were repeated at least three times.

### Drought stress treatment

Three-week-old plants under normal growth condition were fully irrigated, then irrigation was stopped for two weeks. After that, plants were fully irrigated again; then in the next two weeks, no irrigation was applied. After two rounds of drought treatment, plants were harvested. The seed yields, silique numbers, and heights of plants were measured. Three technical repeats with three biological replicates were used.

### Heat stress treatment

Heat tolerance test was conducted on MS plates. Arabidopsis seeds were surface sterilized as described above and sowed on MS plates, and left at 4 °C for three days for stratification, then kept at 32 °C to grow vertically for ten days. After that, root lengths were measured. In addition, the heat tolerance test was conducted with soil grown plants. In this assay, three weeks old plants grown in soil under normal growth condition (i.e., 25 °C and regular irrigation) were transferred into a growth chamber that was set at 37 °C for 5.5 hours per day until the end of the experiment, and seed yields were analyzed. Plants were irrigated with normal water three times a week during the heat treatment.

### Combined heat and drought stresses

Plants were grown in pots in a growth chamber that was set at a photoperiod of 16 h of light and 8 h of darkness per day at 22 °C with regular irrigation for three weeks. Then, plants were fully irrigated before transferring to a growth chamber that was set with the same photoperiod, but with a different temperature regime: 37 °C for 5.5 h from 12:00 pm to 5:30 pm and 22 °C for the rest of time. The irrigation was reduced to half the amount of water used for control plants every five days. For each experiment, three biological replicates for each genotype were used and experiments were repeated three times.

### Combined heat and salt stresses

Arabidopsis seeds were surface sterilized and sown on MS plates supplemented with 100 mM of NaCl. Plates were kept at 32 °C, and root lengths were measured, and the phenotype was documented after ten days. For plants grown in soil, three weeks old plants were transferred to a growth chamber that was set at 37 °C for 5.5 h and 22 °C for 18.5 h per day for 25 days. Starting the day after transferring, plants were irrigated with 50 mM of NaCl, which was repeated on the third day of treatment, and followed by 100 mM of NaCl on the 6^th^ and the 9^th^ days of treatment. After that, plants were irrigated with regular water every other day until the end of the experiment. Three biological replicates for each genotype were used, and experiments were repeated three times.

### Combined drought and salt stresses

Three weeks old plants grown under normal growth condition were irrigated with half the amount of water used for control plants supplemented with 50 mM of NaCl, which was repeated on the 5^th^ day, then followed by 100 mM of NaCl on the 10^th^ and the 15^th^ days. After that, plants were irrigated with water once every five days until the end of the experiment and seed yields were analyzed. Three biological replicates for each genotype were used, and experiments were repeated three times.

### Combined drought, heat and salt stresses

Plants were grown under normal growth conditions for three weeks, then they were moved to a chamber that was set at 37 °C for 5.5 h and 22 °C for 18.5 h per day, irrigated with half the amount of water used for control plants supplemented with 50 mM NaCl on the 1^st^ and the 4^th^ day, and thereafter the concentration of salt increased to 100 mM NaCl in the second week. This was followed by irrigation with half of the amount of normal water used for control plants twice a week until the end of the experiment. Three biological replicates for each genotype were used, and the experiments were repeated three times.

### Rhizosphere acidification assay

The amount of protons released by the roots of Arabidopsis plants to the total root fresh weight was calculated using the method described by Pizzio *et al*.^51^. Surface sterilized seeds of Arabidopsis were sown on MS plates (pH 5.7) and grown vertically for three days. Thereafter seedlings were transferred to MS plates (pH 6.5) containing 0.004% pH indicator bromocresol purple for 7–10 days. Plates were monitored regularly for color change. The color change from purple to yellow indicates the pH change in the media due to acidification of rhizosphere of plants.

### RNA sequencing and differential gene expression analysis

Comparative transcriptome analysis was performed using Arabidopsis plants grown under combined heat and drought stresses and normal growth conditions. Total RNAs were extracted from WT, *AVP1*-overexpressing, *OsSIZ1*-overexpressing, and one of the *AVP1*/*OsSIZ1* co-overexpressing (i.e., AO3) plants using the Spectrum^TM^ Plant Total RNA kit (Sigma, USA). High-quality RNA samples were used for cDNA library construction using the NEB Next mRNA Library Prep Master Mix Set for Illumina (NEB, USA) in accordance with the manufacturer instruction. The cDNA libraries were then sequenced with the Illumina HiSeq. 2500 platform (Illumina, Inc., San Diego, CA, USA) using paired-end technology. The clean reads from all samples were mapped onto the reference Arabidopsis genome using Tophat2 (version 2.2.5)^63^. Transcript levels were measured using EBSeq software^64^ after normalizing the number of reads with reads per kilo-base of transcript per million mapped reads (RPKM) values from each gene. DEGs were identified by setting a cutoff *p*-value < 0.001 and the false discovery rate (FDR) < 0.01. DEGs with a fold-change >2 were considered as up-regulated, and those with <0.5 were considered as down-regulated.

### Real time quantitative PCR analysis

The validation of DEG data generated from RNA sequencing was carried out with quantitative real time PCR (RT-qPCR). Among those DEGs, we selected 9 DEGs related to heat and/or drought stress response. The primers were designed using Primer Premier 5 (PREMIER Biosoft, CA, USA) (Supplementary Table 2). The reverse transcription was carried out using 1 μg of DNase-treated total RNAs and the SuperScript^TM^ VILO^TM^ Master Mix (Invitrogen, USA). The cDNA templates were amplified with Applied Biosystems 7500 Real-Time PCR detection system and the SYBR Green JumStart^TM^ Taq ReadyMix^TM^ (Sigma, USA). The Arabidopsis *Actin8* was used as the internal reference gene, and the relative transcript level of each gene was calculated using the 2^−ΔΔCt^ method^65^. Three biological replicates were used for all RT-qPCR reactions.

### Statistical analysis

Tukey’s statistical method was performed in all pairwise comparison across different genotypes at the significant level of *p = *0.05.

## Supplementary information

Supplementary Materials
