# Supplementary material for: Co-overexpression of AVP1 and OsSIZ1 in Arabidopsis substantially enhances plant tolerance to drought, salt, and heat stresses
Source: Sci Rep. 2019 May 21;9:7642. doi: 10.1038/s41598-019-44062-0 (PMC6529626; doi:10.1038/s41598-019-44062-0)
Supplement: Supplementary file 1 — Supplementary Materials [file 41598_2019_44062_MOESM1_ESM.pdf]

Supplementary Materials

Co-overexpression of *AVP1* and *OsSIZ1* in Arabidopsis substantially enhances plant tolerance to drought, salt, and heat stresses

Nardana Esmaeili[1], Xiaojie Yang[2], Yifan Cai[1], Li Sun[1], Xunlu Zhu[1], Guoxin Shen[3], Paxton Payton[4], Weiping Fang[2, *], and Hong Zhang[1, *]

[1] Department of Biological Sciences, Texas Tech University, Lubbock, Texas 79409, USA

[2] Economic Crop Research Institute, Henan Academy of Agricultural Sciences, Zhengzhou 450002, China

[3] Zhejiang Academy of Agricultural Sciences, Hangzhou, China

[4] USDA-ARS Cropping Systems Research Laboratory, Lubbock, Texas, USA

* Co-corresponding author: Weiping Fang (hncot@163.com); (Hong Zhang (e-mail: hong.zhang@ttu.edu)

Supplementary figures

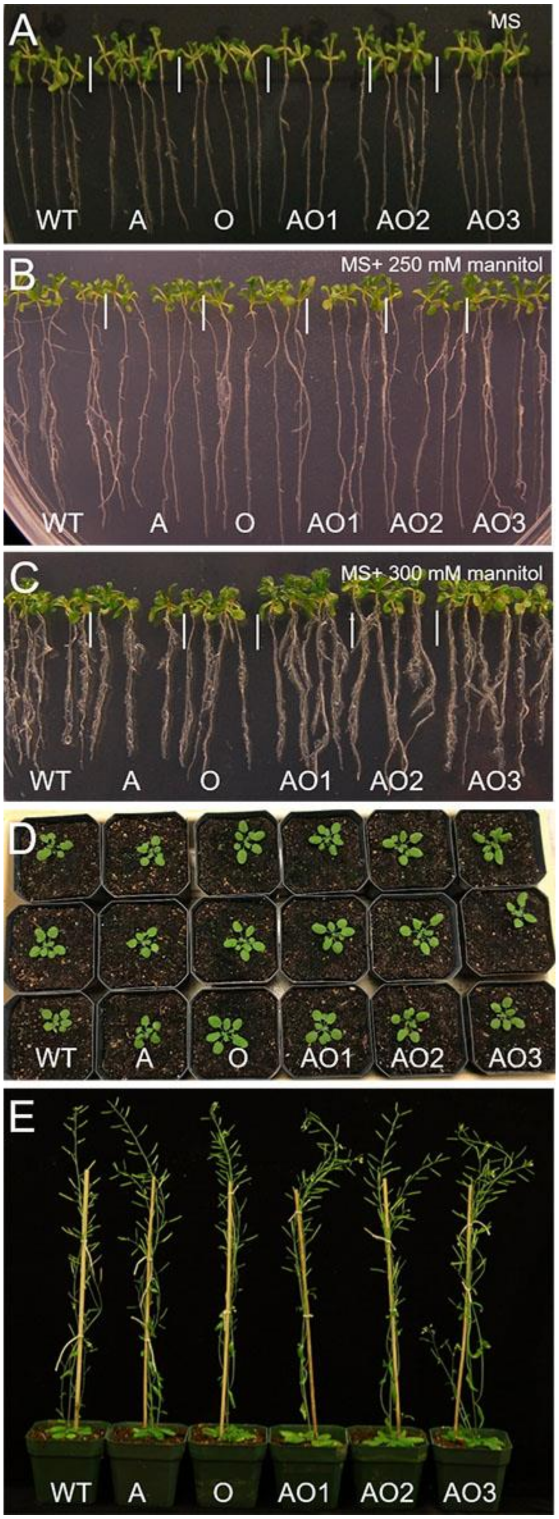

Supp. Fig. 1. Phenotypes of wild-type and transgenic plants on MS plate and in soil under normal growth conditions. **A.** Phenotypes of wild-type and transgenic plants on MS plates. **B.** Phenotypes of wild-type and transgenic plants on MS plates supplemented with 250 mM mannitol. **C.** Phenotypes of wild-type and transgenic plants on MS plates supplemented with 300 mM mannitol. **D.** Phenotypes of three-week-old wild-type and transgenic plants in soil. **E.** Phenotypes of eight-week-old wild-type and transgenic plants in soil. WT, wild-type plant; A, *AVP1*-overexpressing plant; O, *OsSIZ1*-overexpressing plant; AO1 to AO3, three independent *AVP1*/*OsSIZ1* co-overexpressing plants.

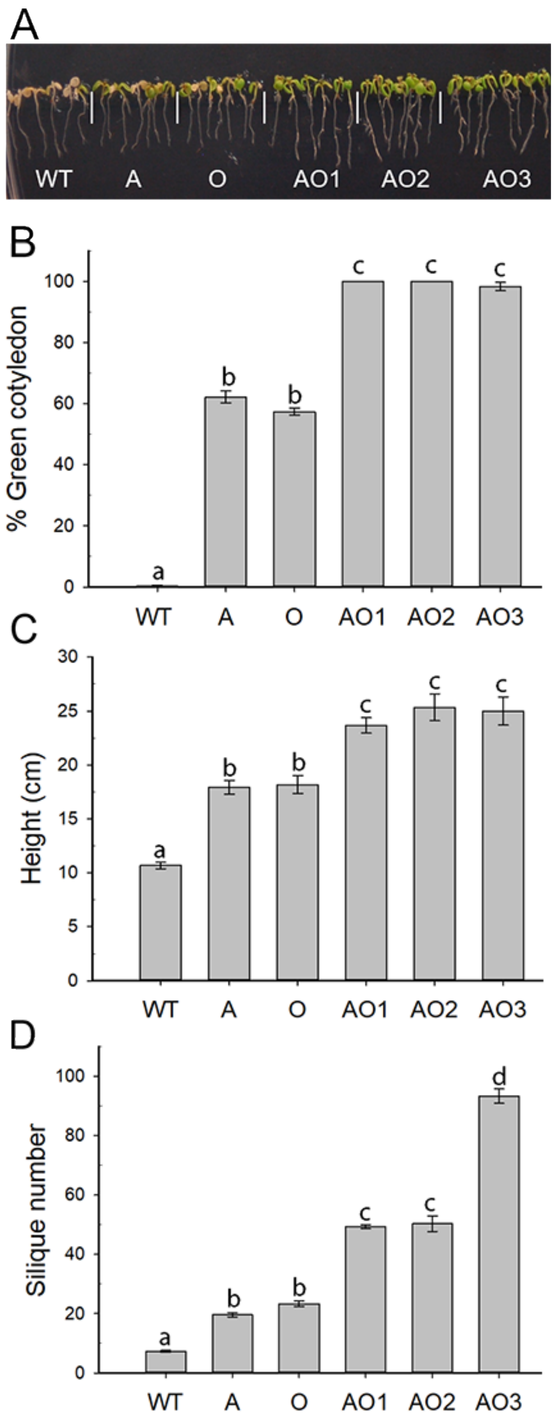

Supp. Fig. 2. Performance of wild-type and transgenic plants under salt stress condition. **A**. Phenotypes of wild-type and transgenic plants on MS plates supplemented with 150 mM NaCl. **B**. Percentage of green cotyledon of wild-type and transgenic plants in A. Data are means ± SE (n = 6). **C**. Plant height of wild-type and transgenic plants under salt stress condition in soil. **D**.

Silique number per plant in wild-type and transgenic plants after salt stress treatment in the soil. Data are means ± SE (n = 9). WT, wild-type plant; A, *AVP1*-overexpressing plant; O, *OsSIZ1*-overexpressing plant; AO1 to AO3, three independent *AVP1*/*OsSIZ1* co-overexpressing plants. Samples denoted by different letters are significantly different ($P < 0.05$, ANOVA, Tukey correction).

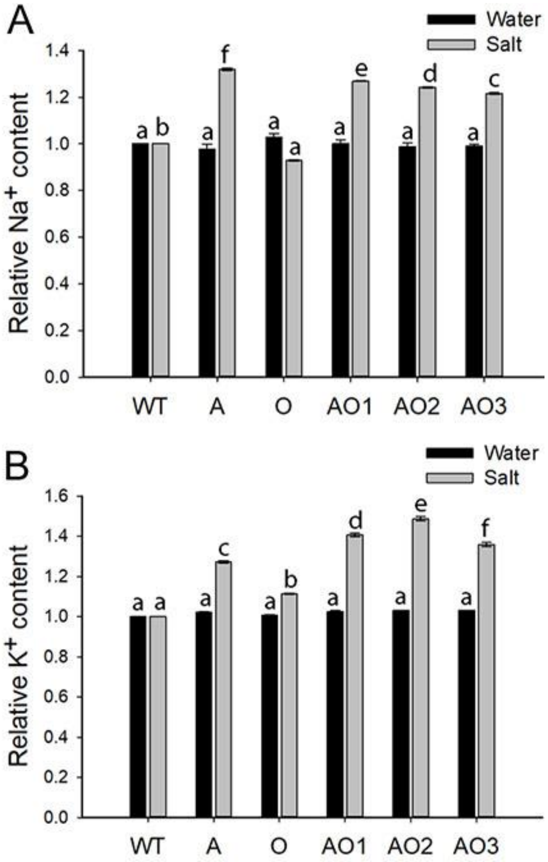

Supp. Fig. 3. Relative contents of sodium and potassium in wild-type and transgenic plants under normal and salt stress conditions. **A.** Relative content of Na^+ in plants growing under normal (dark) and salt (grey) conditions. **B.** Relative content of K^+ in plants growing under normal (dark) and salt (grey) conditions. Data are means ± SE (n = 10). WT, wild-type plant; A, *AVP1*-overexpressing plant; O, *OsSIZ1*-overexpressing plant; AO1 to AO3, three independent *AVP1*/*OsSIZ1* co-overexpressing plants. Samples denoted by different letters are significantly different ($P < 0.05$, ANOVA, Tukey correction).

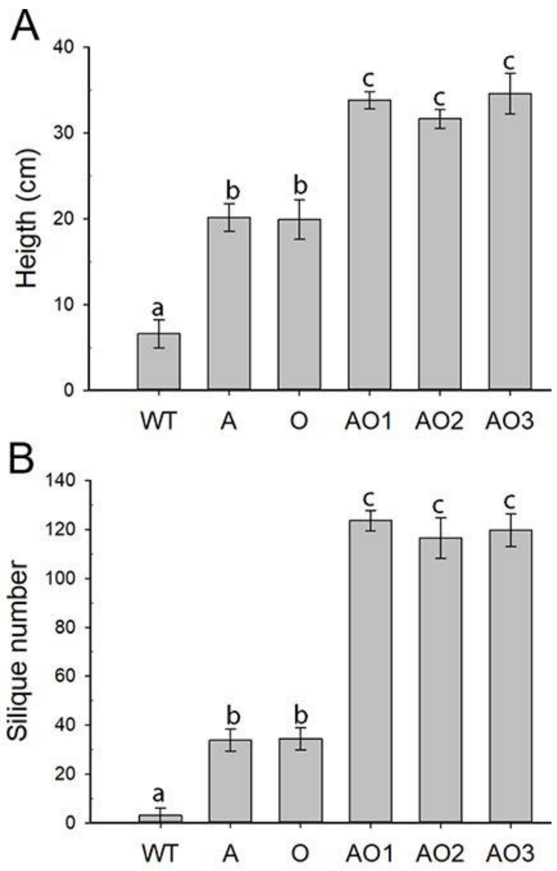

Supp. Fig. 4. Analysis of plant height and silique number in wild-type and transgenic plants under drought stress condition. **A**. Plant height of wild-type and transgenic plants under water deficit treatment. **B**. Silique number per plant in wild-type and transgenic plants after water deficit treatment. Data are means ± SE (n = 9). WT, wild-type plant; A, *AVP1*-overexpressing plant; O, *OsSIZ1*-overexpressing plant; AO1 to AO3, three independent *AVP1*/*OsSIZ1* co-overexpressing plants. Samples denoted by different letters are significantly different ($P < 0.05$, ANOVA, Tukey correction).

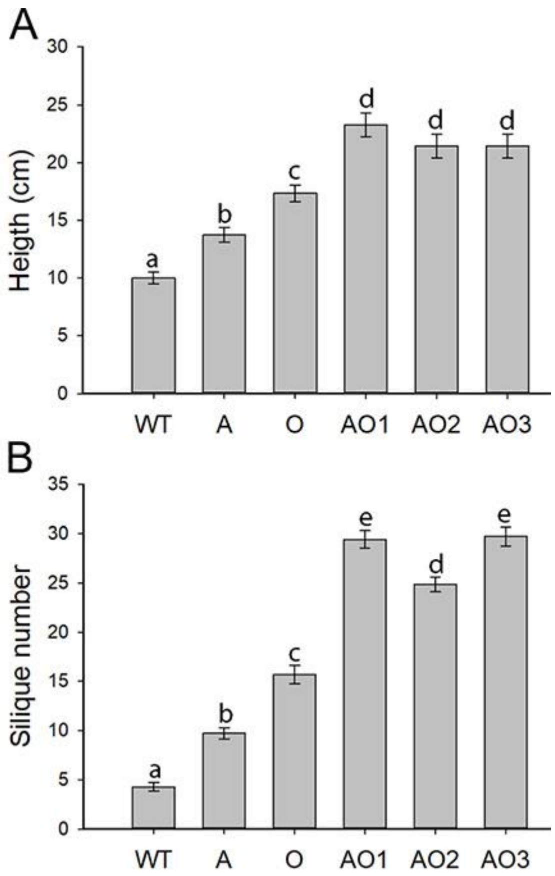

Supp. Fig. 5. Analysis of plant height and silique number in wild-type and transgenic plants under combined heat and salt stresses. **A**. Plant height of wild-type and transgenic plants under combined heat and salt stresses. **B**. Silique number per plant in wild-type and transgenic plants under combined heat and salt stresses. Data are means ± SE (n = 9). WT, wild-type plant; A, *AVP1*-overexpressing plant; O, *OsSIZ1*-overexpressing plant; AO1 to AO3, three independent *AVP1*/*OsSIZ1* co-overexpressing plants. Samples denoted by different letters are significantly different ($P < 0.05$, ANOVA, Tukey correction).

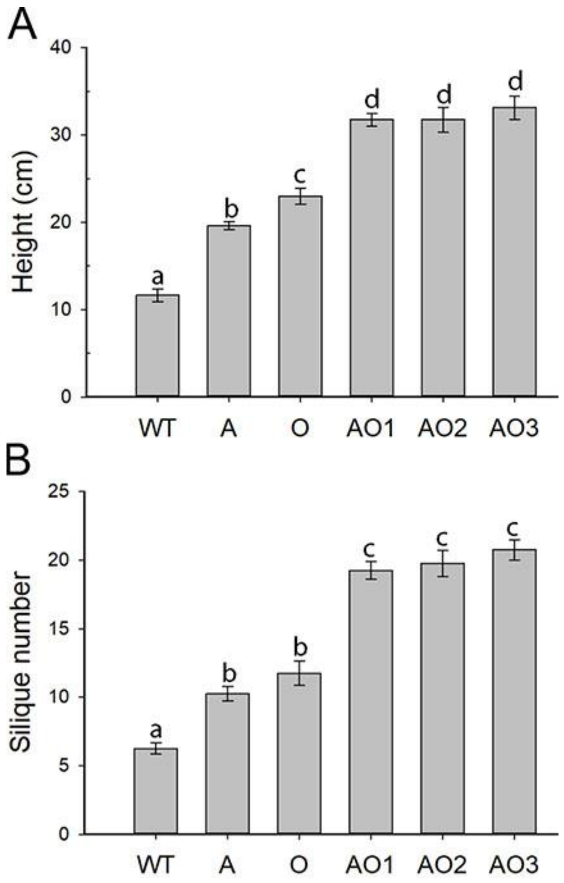

Supp. Fig. 6. Analysis of plant height and silique number in wild-type and transgenic plants under combined heat and drought stresses. **A**. Plant height of wild-type and transgenic plants under combined heat and drought stresses. **B**. Silique number per plant in wild-type and transgenic plants under combined heat and drought stresses. Data are means ± SE (n = 9). WT, wild-type plant; A, *AVP1*-overexpressing plant; O, *OsSIZ1*-overexpressing plant; AO1 to AO3, three independent *AVP1*/*OsSIZ1* co-overexpressing plants. Samples denoted by different letters are significantly different ($P < 0.05$, ANOVA, Tukey correction).

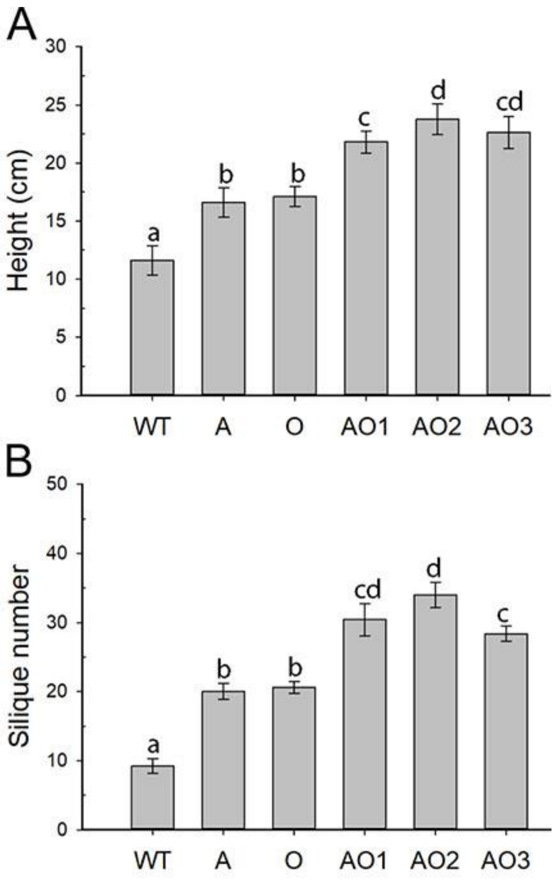

Supp. Fig. 7. Analysis of plant height and silique number in wild-type and transgenic plants under combined salt and drought stresses. **A**. Plant height of wild-type and transgenic plants under combined salt and drought stresses. **B**. Silique number per plant in wild-type and transgenic plants under combined salt and drought stresses. Data are means ± SE (n = 9). WT, wild-type plant; A, *AVP1*-overexpressing plant; O, *OsSIZ1*-overexpressing plant; AO1 to AO3, three independent *AVP1*/*OsSIZ1* co-overexpressing plants. Samples denoted by different letters are significantly different ($P < 0.05$, ANOVA, Tukey correction).

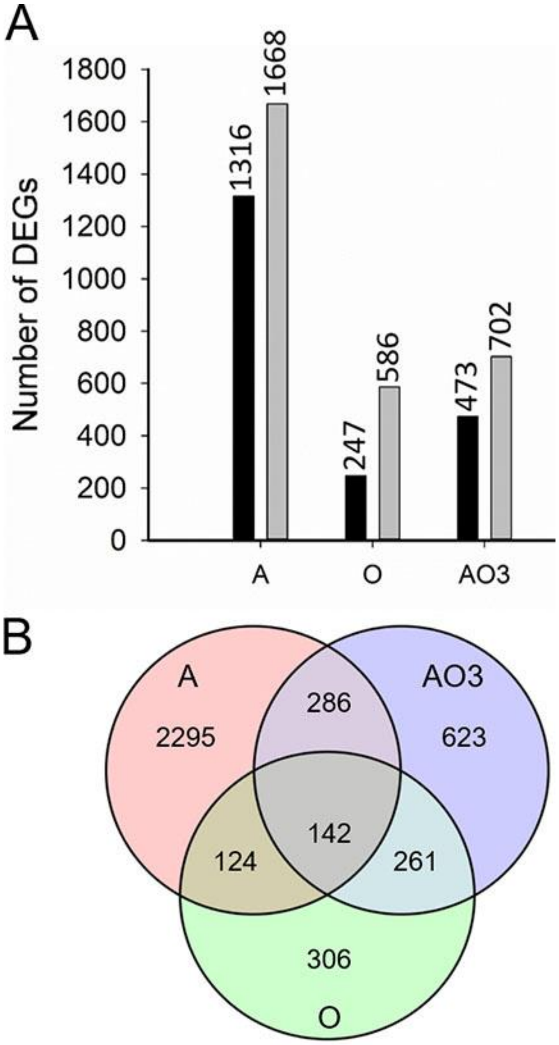

Supp. Fig. 8. RNA sequencing analysis of wild-type, *AVP1*-overexpressing, *OsSIZ1*-overexpressing, and *AVP1*/*OsSIZ1* co-overexpressing plants under normal growth condition. **A**. Number of differentially expressed genes in *AVP1*-overexpressing, *OsSIZ1*-overexpressing, and *AVP1*/*OsSIZ1* co-overexpressing 3 plants vs. wild-type plants under normal growth condition. Black bars represent up-regulated genes, and grey bars represent down-regulated genes. **B**. Venn diagram of differentially expressed genes in *AVP1*-overexpressing, *OsSIZ1*-overexpressing, and *AVP1*/*OsSIZ1* co-overexpressing plants vs. wild-type plants under normal growth condition. DEGs, differentially expressed genes; WT, wild-type plant; A, *AVP1*-overexpressing plant; O, *OsSIZ1*-overexpressing plant; AO3, *AVP1*/*OsSIZ1* co-overexpressing plant 3.

Supplementary Table 1. List of 44 genes whose transcripts were altered in *AVP1/OsSIZ1* co-overexpression plants subjected to treatment of combined heat and drought stresses.

Gene No.	Gene Name
AT2G37180	*RD28*
AT3G14440	*NCED3*
AT2G19590	*ACO1*
AT3G48360	*BT2*
AT4G23810	*WRKY53*
AT4G37260	*MYB73*
AT3G09440	*HSP70-3*
AT5G10140	*FLC*
AT3G46230	*AtHSP17.4*
AT5G56840	*MIK19*
AT5G47600	*HSP14.7*
AT5G67300	*AtMYBR1*
AT1G73330	*AtDR4*
AT2G26150	*AtHSFA2*
AT1G74310	*AtHSP101*
AT3G11020	*DREB26*
AT3G12580	*HSP70*
AT1G52830	*IAA6*
AT5G59720	*HSP18.2*
AT4G34000	*ABF3*
AT1G20450	*ERD10*
AT1G04240	*IAA3*
AT5G52300	*RD29B*
AT1G30360	*ERD4*
AT1G21910	*ERF12*
AT1G71880	*SUC1*
AT4G25480	*DREB1A*
AT4G30190	*AHA2*
AT1G46264	*HSFB4*
AT3G16240	*TIP2*
AT1G52890	*ANAC019*
AT5G40890	*AtCLC-A*
AT2G20880	*ERF053*
AT3G23430	*PHO1*
AT2G23290	*AtMYB70*
AT3G49110	*PER33*
AT2G31230	*AtERF15*
AT2G28190	*CSD2*
AT2G47190	*AtMYB2*
AT3G30775	*ERD5*

AT3G46090	*ZAT7*
AT5G64120	*PER71*

Supplementary Table 2. List of primers used in quantitative real-time PCR analyses

**Gene Name**	**Oligonucleotide Sequence**
DR4:	FP: TGTTCCCTTTCTACTGATTCACAA<br>RP: GACAGTGACACACTGATCATACCA
ERD10:	FP: TGAATAATAATGATGTGGGAGTGG<br>RP: CACAAACTTGGAGAACAGCTAGAA
HSFA2:	FP: CTCAAGTTTTATTCGTCAGCTCAA<br>RP: TGTTCTTCAAGAGATGCTTTTGTC
HSFB4:	FP: AACAACTTCTCTAGCTTCGTTCGT<br>RP: TCTCTCCTCTCTTGAAAAACTCGT
HSP17:	FP: GAGGTCAAGTCCGTTGATATCTCT<br>RP: ACAACGAGATTGGTGATTACAAGA
HSP70:	FP: TTAAGCCTTTTGGCTTTTGTTTAC<br>RP: ATGATCACACACCAGTTTCAGAGT
HSP101:	FP: ACACTCTCTCTCGAATTCTCTGGT<br>RP: GAGGCATGACTGCTTTTGTATATG
NCED101:	FP: ACCTTACCAAGTTCAGATCACTCC<br>RP: GTAGCTTAAAGCGAAGAGTTCACC
RD28:	FP: AACAACCACAAAGCAAAACACTTA<br>RP: TAATCTCTCGTCTGAAATCCATCA
